# PM2.5 Concentration Prediction Model: A CNN–RF Ensemble Framework

**DOI:** 10.3390/ijerph20054077

**Published:** 2023-02-24

**Authors:** Mei-Hsin Chen, Yao-Chung Chen, Tien-Yin Chou, Fang-Shii Ning

**Affiliations:** 1 GIS Research Center, Feng Chia University, Taichung 40724, Taiwan; 2 Department of Land Economics, National Cheng Chi University, Taipei 11605, Taiwan

**Keywords:** PM2.5, convolutional neural network, random forest

## Abstract

Although many machine learning methods have been widely used to predict PM2.5 concentrations, these single or hybrid methods still have some shortcomings. This study integrated the advantages of convolutional neural network (CNN) feature extraction and the regression ability of random forest (RF) to propose a novel CNN-RF ensemble framework for PM2.5 concentration modeling. The observational data from 13 monitoring stations in Kaohsiung in 2021 were selected for model training and testing. First, CNN was implemented to extract key meteorological and pollution data. Subsequently, the RF algorithm was employed to train the model with five input factors, namely the extracted features from the CNN and spatiotemporal factors, including the day of the year, the hour of the day, latitude, and longitude. Independent observations from two stations were used to evaluate the models. The findings demonstrated that the proposed CNN–RF model had better modeling capability compared with the independent CNN and RF models: the average improvements in root mean square error (RMSE) and mean absolute error (MAE) ranged from 8.10% to 11.11%, respectively. In addition, the proposed CNN–RF hybrid model has fewer excess residuals at thresholds of 10 μg/m^3^, 20 μg/m^3^, and 30 μg/m^3^. The results revealed that the proposed CNN–RF ensemble framework is a stable, reliable, and accurate method that can generate superior results compared with the single CNN and RF methods. The proposed method could be a valuable reference for readers and may inspire researchers to develop even more effective methods for air pollution modeling. This research has important implications for air pollution research, data analysis, model estimation, and machine learning.

## 1. Introduction

According to the World Health Organization (WHO), air pollution kills nearly 7 million people worldwide every year. Currently, nine out of ten people breathe air that exceeds WHO guidelines for pollutants, with those living in low- and middle-income countries suffering the most [1]. Pollutants that are major public health problems include particulate matter, carbon monoxide, ozone, nitrogen dioxide, sulfur dioxide, black carbon, and polycyclic aromatic hydrocarbons. Many scholars have suggested that air pollutants can cause considerable damage to humans and the living environment [2,3,4,5,6,7,8,9].

Ambient particulate matter (PM), a primary type of air pollution, consists of small solid or liquid particles suspended in the air; therefore, it is also called atmospheric PM. Generally, suspended particles with an aerodynamic diameter of less than or equal to 2.5 µm are classified as fine particulate matter (PM2.5) [10]; those with an aerodynamic diameter less than or equal to 10 µm are classified as particulate matter (PM10). As early as the 1970s, studies indicated a relationship between suspended particulates and human health. Multiple studies have verified that aerosols cause diseases of the respiratory and cardiovascular systems, which may lead to conditions such as asthma, lung cancer, birth defects, and premature death [11]. Therefore, governments worldwide are currently striving to effectively manage and control PM levels. Due to the high impact of PM2.5 concentration on human health and environmental ecology, an accurate, robust, and reliable prediction model of PM2.5 concentration could provide governments and the people with prior prevention and control of air pollution.

The methods for predicting PM2.5 concentrations can generally be divided into physical and statistical methods [12]. Physical methods employ the principles of physics, chemistry, meteorology, and biology to predict PM2.5 concentrations; researchers implement simulations to analyze the emission, diffusion, conversion, and removal processes of PM2.5. The representative models of PM2.5 concentrations based on physical methods include the following: the Community Multiscale Air Quality Model [13,14], the Nested Air Quality Prediction Modeling System [15,16], the Weather Research and Forecasting Model with Chemistry [17,18], and Satellite Data Inversion [16,19,20]. However, the aforementioned models generally use a known theoretical model to describe the relationship between factors to estimate PM2.5 concentrations. Complex theoretical models require a large amount of observation data and a long testing period. Therefore, the disadvantages of physical methods include their consumption of resources and manpower. In contrast to physical methods, statistical methods do not require a predetermined theoretical model; they employ black box models that use investigations and analyses from historical pollutant data to generate prediction results [21]. Statistical methods, including machine learning and deep learning [22], have been widely employed to predict PM2.5 concentrations. Machine learning methods and deep learning methods comprise the current mainstream models [23].

With advancements in computer technology, machine learning methods are increasingly employed in the prediction of PM2.5 concentrations. Classical machine learning methods that have demonstrated good predictive ability for PM2.5 concentrations include decision trees (DTs) [24,25], random forest (RF) [26,27,28], support vector machine (SVM) [29,30,31], and artificial neural networks (ANNs) [32,33,34]. Deep learning, a subtype of machine learning, can solve many problems of traditional machine learning, such as slow convergence speed, insufficient generalization ability, and the influence of irrelevant features on the prediction results. In addition, deep learning methods can generate more robust predictions and better approximate nonlinear data. Therefore, in recent years, PM2.5 concentration prediction models have widely employed deep learning methods. Types of deep learning methods include convolutional neural networks (CNNs), which can extract the key information from inputs; recurrent neural networks (RNNs), which effectively process data with sequence features, such as time and semantic information; long short-term memory (LSTM) networks, which can process and predict critical events with long intervals and delays in time-series data; and bidirectional LSTM (BiLSTM) networks, which connect two hidden layers and operate bidirectionally between inputs and outputs. Researchers have employed the deep learning methods of CNN [35,36,37], RNN [38,39,40], LSTM [39,41,42], and Bi-LSTM [43,44] to predict pollutant concentrations.

Hybrid machine learning models have been widely employed in pollutant concentration prediction research because they are able to better quantify complex data. Popular models include multiple linear regression–autoregressive integrated moving average (MLR–ARIMA) [45], empirical mode decomposition–convolutional neural network (EMD–CNN) [46,47], long short-term memory–fully connected (LSTM-FC) neural network [48], extreme gradient boosting–multi-scale convolutional neural network–genetic algorithm–long short-term memory (XGBoost–MSCNN–GA–LSTM) [49], CNN–LSTM [50,51,52], CNN–BiLSTM [53], and convolutional block attention module–convolutional neural network–bidirectional LSTM (CBAM–CNN–BiLSTM) [54,55]. However, the aforementioned models face limitations; some only apply to a single station, those with complex structures require more resources, and others do not incorporate spatiotemporal factors.

Machine learning methods typically use a variety of factors to predict PM2.5 concentrations, including meteorological, air pollution, spatiotemporal, land use, and satellite remote sensing data [56,57,58,59,60]. The factors selected depend on the modeling goals and available data. In this study, the focus was on developing a method for multistation PM2.5 concentration prediction models. To ensure data acquisition convenience, meteorological and air pollution parameters from ground air quality monitoring stations, along with their corresponding spatiotemporal parameters, were used for modeling. The CNN convolutional layer is used to extract key features through deep learning techniques. The RF machine learning technique has shown good predictive accuracy, stability, and speed. Therefore, this study proposed a hybrid method, CNN–RF, that combines deep learning with machine learning. This design exploits the advantages of each method to construct a stable, reliable, and accurate prediction model for PM2.5 concentrations.

This paper is organized as follows: Section 2 introduces the related algorithms and experiments employed in this study and describes the construction of the proposed CNN–RF framework. Section 3 presents an analysis and comparison of the research results. Section 4 states the conclusion and recommendations for future research.

## 2. Datasets and Methods

### 2.1. Datasets and Preprocessing

**Figure 1:**
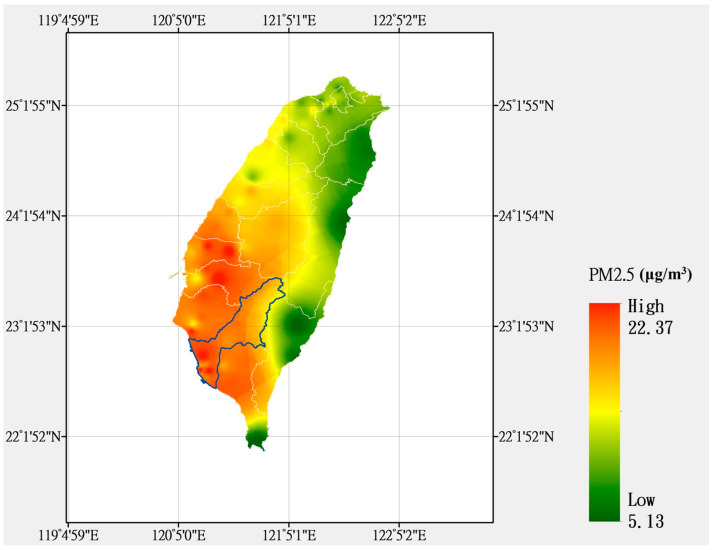
Distribution of annual average PM2.5 concentration from 2021 in Kaohsiung, Taiwan.

This study selected the industrial city of Kaohsiung as the research area due to its persistent air pollution. According to observation data from the Taiwan Air Quality Monitoring Network, Kaohsiung had the highest annual average PM2.5 concentrations in Taiwan in 2021 (Figure 1). This study used the observation data collected by 13 monitoring stations in the Kaohsiung area in 2021 to construct a PM2.5 concentration model; the data, which included hourly weather conditions, air pollution values, location, and time, were downloaded from the Taiwan Air Quality Monitoring Network of the Environmental Protection Administration. The observation data from 11 monitoring stations were employed for model training. To avoid overfitting, which could possibility affect the model’s performance and verify the generalization ability of the training model, independent observation data from two monitoring stations in Fuxing and Fongshan were used for model testing.

**Figure 2:**
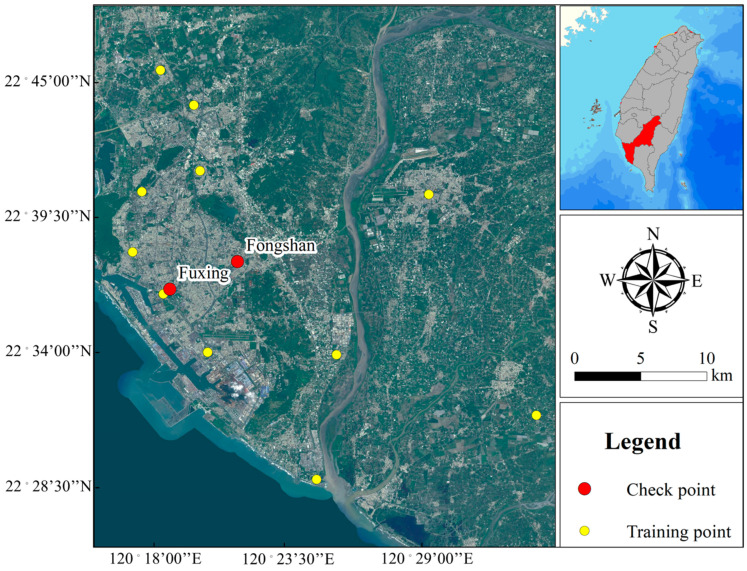
Distribution diagram of training and independent testing stations.

The testing stations used in this study are part of the Taiwan Air Quality Monitoring Network’s six traffic air quality monitoring stations. These stations are strategically located in areas with high traffic flow or high pollution caused by traffic emissions, both of which are prevalent in the prosperous areas of Kaohsiung. Fongshan, one of the testing station’s locations, is the most populous administrative district in Kaohsiung. In terms of geographical location, the Fuxing station is located adjacent to the training stations, while the Fongshan station is positioned in the middle of the training stations, making it farther away from the training stations than the Fuxing station. The testing data consist of 15,943 observations, which is 18% of the training data, meeting the necessary requirements for model testing. Therefore, the selected testing stations are in high traffic flow areas with a dense population and high land use, which are factors that significantly impact PM2.5 concentration. The difference in distance between the testing stations and the adjacent training stations allows for an examination of the modeling performance influenced by the spatial factor (Figure 2).

This study collected the hourly observation data of 13 monitoring stations from 1 January to 31 December 2021. Excluding missing data, this study collected 88,383 training observations and 15,943 independent testing observations, including 8018 and 7925 observations from the Fuxing and Fongshan stations, respectively (Table 1). Each observation was composed of eight air pollution factors, specifically CO, NO_2_, NO, NO_X_, SO_2_, O_3_, PM10, and PM2.5; five meteorological factors, which include wind speed, wind direction, relative humidity, rainfall, and ambient temperature, as well as four spatiotemporal factors, namely day of the year (DoY), hour of the day (HoD), latitude (Lat), and longitude (Long). This study aims to develop a new model for predicting PM2.5 concentration. Given the air pollution and meteorological factors at a particular location and time, the model estimates the unknown PM2.5 concentration. However, the focus of the study is not on examining the relationship between PM2.5 concentration and air pollution or meteorological factors. Therefore, the PM2.5 concentration collected is treated as the dependent variable in the proposed CNN-RF model, and the other factors are treated as independent variables.

Because the independent variables did not contribute equally to the model fitting, bias could occur. Min–max normalization, one of the most common methods to normalize data, was implemented for the independent variables. For each variable, the minimum value is transformed into 0, the maximum value is transformed into 1, and every other value is transformed into a value between 0 and 1. This method is able to improve the convergence speed and accuracy of the model. The conversion method (Equation (1)) is expressed as follows:

(1) $$X_{nom}=\frac{X-X_{\min}}{X_{\max}-X_{\min}}\in \left[0,1\right]\;\;$$

where $X_{nom}$ is the normalized independent variable, X is the original independent variable, $X_{\min}$ is the minimum value of the original independent variable, and $X_{\max}$ is the maximum value of the original independent variable.

### 2.2. Proposed CNN–RF Framework

CNN is a class of ANN for processing data with a grid-like pattern. It employs a convolutional deep learning technique to achieve feature extraction; features are automatically deduced and optimally tuned for the desired outcome. CNN has a mathematical structure that typically consists of three layers, which include the convolutional layer, the pooling layer, and the fully connected layer. The first two convolutional layers and the pooling layer extract features (i.e., feature learning), whereas the fully connected layer maps the extracted features for the final output. The convolutional layer, which plays a key role in CNN, completes multiple mathematical operations—these include convolution, a special type of linear unit. CNN can efficiently process images; therefore, it is commonly employed to analyze visual images, which includes tasks such as image classification, segmentation, medical image analysis, and natural language processing. The flexible nature of deep learning enables its adaptation to process time-series data.

RF, a prediction modeling and behavioral analysis technique, is based on DTs. It employs bagging or bootstrap aggregation, an ensemble learning technique. The RF method fits many DTs on subsamples of the data set and combines the output of all the DTs. This method achieves greater accuracy because it is able to reduce the problems of variance and overfitting in DTs. The RF technique considers individual instances and uses the instance that receives the most votes as its prediction. Each tree receives inputs from samples in the initial dataset. Features are then randomly selected, which are used to generate nodes on each tree. The trees in the forest should not be pruned until a decisive forecast is reached at the end of the exercise. Thus, RF enables any classifier with weak correlations to create a strong classifier. The RF technique also employs an advanced method to address missing data. Missing values are replaced by the variable that occurs most often in a particular node. Because this method processes variables quickly, it is well-suited for complicated tasks. The RF method exhibits outstanding predictive ability.

**Figure 3:**
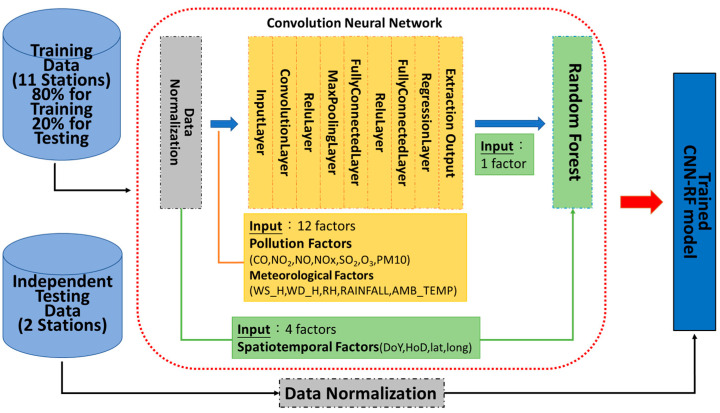
Architecture of the proposed CNN–RF method.

As mentioned in the Introduction, hybrid methods have been widely employed in PM2.5 concentration prediction research because they are able to better quantify complex data. However, not all models can be effectively combined—which methods are suitable for ensemble and processes in the data processing needs to be studied. The proposed CNN–RF model in this study exploits the advantages of CNNs and the RF method. A CNN was implemented to extract features from air pollution and meteorological data. This study employed the extracted feature (i.e., the predicted PM2.5) and spatiotemporal variables from each observation, such as DoY, HoD, Lat, and Long, as the input data and PM2.5 as the output data; the RF method was implemented to construct the model of PM2.5 concentrations (Figure 3). Table 2 presents the hyperparameters of the proposed CNN–RF, CNN, and RF models. 

### 2.3. Experimental Equipment and Assessment Indicators

This study employed the Acer (ASUSTeK computer) ExpertCenter D700SC_M700MC and Matlab R2022b with Microsoft Windows 10 Professional Edition. The model verification process was divided into training and independent testing. The mean square error (MSE), root mean square error (RMSE), mean absolute error (MAE), coefficient of determination, (R^2^), and mean accuracy (MA) were employed to evaluate model performance. In addition, the numbers of large residuals were calculated to assess model reliability and stability. The residual threshold was set to 10 μg/m^3^, 20 μg/m^3^, and 30 μg/m^3^, respectively. The relevant assessment indicators are expressed as follows (Equations (2)–(6)):

(2) $$\mathrm{MSE}=\frac{\sum_{i=1}^{n}{\left(y_{\mathrm{tru},i}-y_{p,i}\right)}^{2}}{n}\;$$

(3) $$\mathrm{RMSE}=\sqrt{\frac{\sum_{i=1}^{n}{\left(y_{\mathrm{tru},i}-y_{p,i}\right)}^{2}}{n}}$$

(4) $$\mathrm{MAE}=\frac{\sum_{i=1}^{n}\left|y_{p,i}-y_{\mathrm{tru},i}\right|}{n}\;$$

(5) $$R^{2}=1-\frac{\sum_{i=1}^{n}{\left(y_{\mathrm{tru},i}-\bar{y}\right)}^{2}}{\sum_{i=1}^{n}{\left(y_{\mathrm{tru},i}-y_{p,i}\right)}^{2}}$$

(6) $$\mathrm{MA}=1-\sum_{i=1}^{n}\frac{\left|y_{p,i}-y_{\mathrm{tru},i}\right|}{y_{\mathrm{tru},i}}/n$$

where $y_{\mathrm{tru},i}$ is the actual measured value of the ith PM2.5 concentration, $y_{p,i}$ is the estimated concentration of the ith PM2.5, n is the number of observations, and $\bar{y}$ is the mean of the actual measured value.

## 3. Results and Discussion

### 3.1. Model Evaluation

This section compares the PM2.5 modeling performances of the CNN–RF, CNN, and RF models. The performance evaluation indicators for model training are presented in Table 3. The indicators include calculations for 88,383 hourly predicted PM2.5 concentrations and 88,383 hourly PM2.5 observations. The results reveal that the RF method had the best training performance, followed by the CNN–RF model. The RMSE, MSE, MAE, and R^2^ of the CNN–RF model were 3.67 μg/m^3^, 13.44 μg/m^3^, 2.66 μg/m^3^, and 0.93, respectively. All the evaluation indicators of the CNN–RF model were similar to those of the RF method. Both the RF and CNN–RF models achieved good training results. 

**Figure 4:**
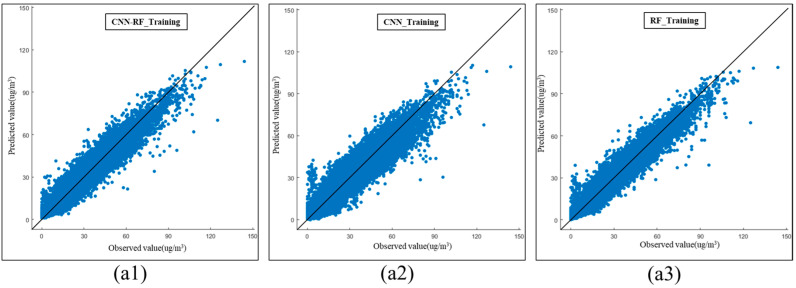
Scatterplots of predictive PM2.5 concentration models. (**a1**) Prediction scatterplot of CNN–RF model with training data. (**a2**) Prediction scatterplot of CNN method with training data. (**a3**) Prediction scatterplot of RF method with training data.

Figure 4 illustrates the scatterplots of the predictive PM2.5 concentration models. The RF method generated good predictions of PM2.5 concentration values during model training, following the proposed CNN–RF model. 

A good prediction model is one that is accurate and reliable in its predictions. In addition to accuracy, a reliable prediction model should also be robust and able to generalize well to new data. This means that the model should not only perform well on the training data it was trained on but also on new data that it has not seen before. The model’s consistency ability for training and validation is an important indicator for assessing a model. Generally speaking, the training accuracy of the model will be better than the validation accuracy, but if the difference is too large, it will lead to an overfitting situation, which can easily result in the overestimation and misjudgment of the model. The further validation of the model’s performance was conducted using an independent testing set. This allowed for an unbiased evaluation of the model’s ability to generalize to new, unseen data, which is an important step in machine learning model development and evaluation.

### 3.2. Model Validation

Independent testing assessed the capability of the three models. Table 4 presents the results for the three models at the Fuxing and Fongshan testing stations. The results differ from those of the training. The proposed CNN–RF model had the best performance for all assessment indicators at the two testing stations; the RMSE, MAE, R^2^, and MA at the Fuxing station were 4.69 μg/m^3^, 3.47 μg/m^3^, 0.89, and 83.81, respectively. At the Fongshan station, the RMSE, MAE, R^2^, and MA were 5.06 μg/m^3^, 3.79 μg/m^3^, 0.88, and 83.50, respectively. Notably, all models had better Mas at the Fuxing station than at the Fongshan station. A possible explanation for this phenomenon is that the Fuxing station is closer to the training station than the Fongshan station is (Figure 2).

This study calculated the average values of RMSE and MAE at the Fuxing and Fongshan stations to compare the performance of the proposed CNN–RF method with those of the RF and CNN methods, as presented in Table 5. The average values of the CNN–RF model for RMSE, MAE, R^2^, and MA were 4.88 μg/m^3^, 3.63 μg/m^3^, 0.88, and 83.66, respectively. Compared with RF and CNN, the CNN–RF model had an RMSE improvement rate of 8.61% and 11.11%, respectively. Furthermore, compared with RF and CNN, the CNN–RF model had an MAE improvement rate of 8.10% and 9.48%, respectively. The findings demonstrate that the CNN–RF model had the best testing performance.

This study estimated the quantities of residuals exceeding the thresholds of 10 μg/m^3^, 20 μg/m^3^, and 30 μg/m^3^ to assess the model’s stability and reliability. The results in Table 6 reveal a phenomenon similar to the aforementioned results. The RF method had fewer excess residuals at each threshold during model training, followed by the CNN–RF model. The number of excess residuals at thresholds of 10 μg/m^3^, 20 μg/m^3^, and 30 μg/m^3^ were 1212, 72, and 14, respectively. The CNN–RF and RF models had similar numbers of excess residuals. The proposed CNN–RF model yielded the best results at the testing stations; the quantities of excess residuals were lower than those of the RF and CNN methods for all stations and thresholds. At the Fuxing station, the proposed model had excess residuals of 344, 14, and 0 at thresholds of 10 μg/m^3^, 20 μg/m^3^, and 30 μg/m^3^, respectively; at the Fongshan station, the excess residuals were 423, 21, and 1 at thresholds of 10 μg/m^3^, 20 μg/m^3^, and 30 μg/m^3^, respectively.

**Figure 5:**
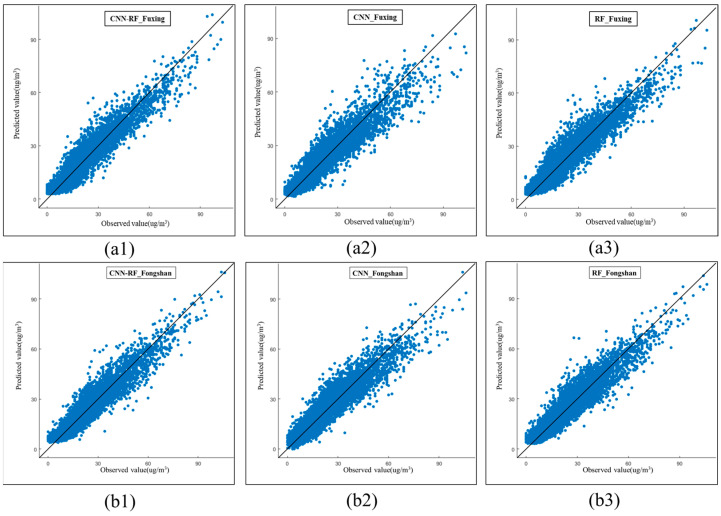
Scatterplots of predictive PM2.5 concentration models. (**a1**) Prediction scatterplot of CNN–RF model at the Fuxing station. (**a2**) Prediction scatterplot of CNN method at the Fuxing station. (**a3**) Prediction scatterplot of RF method at the Fuxing station. (**b1**) Prediction scatterplot of CNN–RF model at the Fongshan station. **(b2**) Prediction scatterplot of CNN method at the Fongshan station. (**b3**) Prediction scatterplot of RF method at the Fongshan station.

Figure 5 illustrates the scatterplots of the predictive PM2.5 concentration models. The proposed CNN–RF model performed well during independent model testing. The proposed CNN–RF model yielded stable, reliable, and accurate results for PM2.5 concentration modeling. The PM2.5 prediction research field is able to successfully adopt this hybrid method. 

The results of this research show that RF has a large difference in training and validation results in RMSE, MAE, and R^2^, while CNN-RF performs better than RF and has better validation results. This suggests that the CNN-RF proposed in this study is accurate, robust, and reliable. The CNN-RF model combines the feature learning and spatial relationship abilities of the CNN with the ensemble learning and averaging capabilities of the RF. The advantage of the CNN-RF model is that it effectively combines the strengths of both CNN and RF methods, resulting in improved performance in PM2.5 concentration modeling. This hybrid approach can potentially be applied to a range of prediction tasks and provide useful insights for researchers in the fields of air pollution, data analysis, model estimation, and machine learning.

## 4. Conclusions

This study proposed a novel method, CNN-RF, for predicting PM2.5 concentrations by combining the advantages of CNN feature extraction and RF regression. The method involves using CNN to extract key meteorological and pollution data and reducing it to a single key factor, which is then combined with spatiotemporal factors and used with RF to construct a PM2.5 concentration prediction model. The proposed CNN-RF was tested on observational data from 13 monitoring stations in Kaohsiung, Taiwan, in 2021. The results showed that the proposed CNN-RF outperformed both the independent CNN and RF models. A summary of the results is given in the following sections.

### 4.1. Model Evaluation

Surprisingly, the model training performance of the proposed CNN-RF model was suboptimal. Instead, the RF model exhibited excellent performance with an R^2^ value of 0.94. Notably, the RF model outperformed the CNN-RF model by approximately 9% and 11% in terms of RMSE and MAE, respectively, indicating its strong ability to fit the training data. However, this study reinforces the importance of utilizing an independent validation mechanism in experiments to determine the presence of overfitting, as model evaluation should not solely rely on training results. The results demonstrate that the CNN-RF model effectively mitigated overfitting.

### 4.2. Model Validation

Once again, the proposed CNN-RF model surprised us by demonstrating superior validation performance, despite having lower training performance than the RF model. In terms of RMSE and MAE, the CNN-RF model outperformed the RF model by approximately 8%. When evaluating the model’s training and validation performance together, the proposed CNN-RF model showed a remarkable improvement of almost 20%, from being approximately 10% lower than the RF model to being 8% better than it. This phenomenon is rare in a predictive model, indicating the effectiveness of the proposed integrated framework, which combines the strengths of CNN and RF. The proposed model not only demonstrated the best performance during model validation but also exhibited minimal differences between training and validation results, demonstrating consistency and generalizability, which are important indicators of a good model. Therefore, the proposed CNN-RF model possesses these desirable characteristics and represents a significant advancement in the field.

In terms of future research, it would be interesting to test the proposed CNN-RF model on a national scale rather than just focusing on the urban area of Kaohsiung. Additionally, integrating LSTM for the early forecasting of PM2.5 concentrations could be a promising direction for further investigation.

## Figures and Tables

**Table 1:** Number of observations for training and testing.

Number of Observations	Training	Testing	
(CO, NO_2_, NO, NO_X_, SO_2_, O_3_, PM10, PM2.5, wind speed, wind direction, relative humidity, rainfall, ambient temperature, day of year, hour of day, latitude, longitude)		Fuxing	Fongshan
	88,383	8018	7925

**Table 2:** Hyperparameters of the proposed CNN–RF model.

Parameters	Value
Convolution layer	1
Kernel size of CNN	3 × 1
Convolution layer channels	16
Max pooling layer	1
Pooling filter size, stride	2 × 1, 2
Fully connected layer	1
Fully connected nodes	384
Learning rate	0.005
Batch size	16
Epochs	20
Mini leaf size	8
Number of learners	30

**Table 3:** Performance evaluation indicators for model training.

Method	RMSE (μg/m^3^)	MSE (μg/m^3^)^2^	MAE (μg/m^3^)	R^2^
RF	3.36	11.27	2.38	0.94
CNN	4.65	21.66	3.43	0.89
CNN-RF	3.67	13.44	2.66	0.93

**Table 4:** Model performance at two testing stations.

Method	RMSE(μg/m^3^)		MAE(μg/m^3^)		R^2^		MA(%)	
	Fuxing	Fongshan	Fuxing	Fongshan	Fuxing	Fongshan	Fuxing	Fongshan
RF	4.93	5.75	3.61	4.30	0.88	0.84	83.20	81.33
CNN	5.54	5.43	3.95	4.08	0.83	0.85	82.26	81.61
CNN-RF	4.69	5.06	3.47	3.79	0.89	0.88	83.81	83.50

**Table 5:** Model comparison with average indicators at two testing stations.

Method	RMSE (μg/m^3^)		MAE (μg/m^3^)		R^2^	MA (%)
	Average	CNN-RF Improvement Rate (%)	Average	CNN-RF Improvement Rate (%)	Average	
RF	5.34	8.61	3.95	8.10	0.86	82.26
CNN	5.49	11.11	4.01	9.48	0.84	81.94
CNN-RF	4.88	---	3.63	---	0.88	83.66
Improvement Rate (%) = (Method − CNN − RF)/Method × 100						

**Table 6:** Excess residuals by model.

Method	>10			>20			>30 (μg/m^3^)		
	Training	Fuxing	Fongshan	Training	Fuxing	Fongshan	Training	Fuxing	Fongshan
RF	1212	418	476	72	26	23	14	2	2
CNN	3501	583	536	213	52	36	33	9	1
CNN-RF	1595	344	423	76	14	21	15	0	1

## Data Availability

Not applicable.

## References

[1] World Health Organization. https://www.who.int/health-topics/air-pollution#tab=tab_1.

[2] Bailie C.R., Ghosh J.K.C., Kirk M.D., Sullivan S.G. (2022). Effect of ambient PM2.5 on healthcare utilisation for acute respiratory illness, Melbourne, Victoria, 2014–2019. J. Air Waste Manag. Assoc..

[3] Syuhada G., Akbar A., Hardiawan D., Pun V., Darmawan A., Heryati H.A., Siregar A.Y.M., Kusuma R.R., Driejana R., Ingole V. (2023). Impacts of Air Pollution on Health and Cost of Illness in Jakarta, Indonesia. Int. J. Environ. Res. Public Health.

[4] Cocârţă D., Prodana M., Demetrescu I., Lungu P., Didilescu A. (2021). Indoor air pollution with fine particles and implications for workers’ health in dental offices: A brief review. Sustainability.

[5] Yang J., Zhang B. (2018). Air pollution and healthcare expenditure: Implication for the benefit of air pollution control in China. Environ. Int..

[6] Kurwadkar S., Sankar T.K., Kumar A., Ambade B., Gautam S., Gautam A.S., Biswas J.K., Salam M.A. (2023). Emissions of black carbon and polycyclic aromatic hydrocarbons: Potential implications of cultural practices during the COVID-19 pandemic. Gondwana Res..

[7] Ambade B., Sethi S.S., Kurwadkar S., Mishra P., Tripathee L. (2022). Accumulation of polycyclic aromatic hydrocarbons (PAHs) in surface sediment residues of Mahanadi River Estuary: Abundance, source, and risk assessment. Mar. Pollut. Bull..

[8] Ambade B., Sankar T.K., Kumar A., Gautam A.S., Gautam S. (2021). COVID-19 lockdowns reduce the Black carbon and polycyclic aromatic hydrocarbons of the Asian atmosphere: Source apportionment and health hazard evaluation. Environ. Dev. Sustain..

[9] Ambade B., Sankar T.K., Sahu L.K., Gautam S., Gautam A.S. (2021). Comparison of emission profile of black carbon and carbon monoxide over Eastern India: Source apportionment and health risk impact. Environ. Sci..

[10] Hinds W.C.A.T. (1999). Introduction.

[11] Zhang M., Mueller N.T., Wang H., Hong X., Appel L.J., Wang X. (2018). Maternal exposure to ambient particulate matter≤ 2.5 µm during pregnancy and the risk for high blood pressure in childhood. Hypertension.

[12] Park S., Kim M., Kim M., Namgung H.-G., Kim K.-T., Cho K.H., Kwon S.-B. (2018). Predicting PM10 concentration in Seoul metropolitan subway stations using artificial neural network (ANN). J. Hazard. Mater..

[13] Djalalova I., Delle Monache L., Wilczak J. (2015). PM2.5 analog forecast and Kalman filter post-processing for the Community Multiscale Air Quality (CMAQ) model. Atmos. Environ..

[14] Woody M., Wong H.-W., West J., Arunachalam S. (2016). Multiscale predictions of aviation-attributable PM2.5 for US airports modeled using CMAQ with plume-in-grid and an aircraft-specific 1-D emission model. Atmos. Environ..

[15] Zhu B., Akimoto H., Wang Z. The Preliminary Application of a Nested Air Quality Prediction Modeling System in Kanto Area, Japan. Proceedings of the AGU Fall Meeting Abstracts.

[16] Geng G., Zhang Q., Martin R.V., van Donkelaar A., Huo H., Che H., Lin J., He K. (2015). Estimating long-term PM2.5 concentrations in China using satellite-based aerosol optical depth and a chemical transport model. Remote Sens. Environ..

[17] Saide P.E., Carmichael G.R., Spak S.N., Gallardo L., Osses A.E., Mena-Carrasco M.A., Pagowski M. (2011). Forecasting urban PM10 and PM2.5 pollution episodes in very stable nocturnal conditions and complex terrain using WRF–Chem CO tracer model. Atmos. Environ..

[18] Wang P., Qiao X., Zhang H. (2020). Modeling PM2.5 and O3 with aerosol feedbacks using WRF/Chem over the Sichuan Basin, southwestern China. Chemosphere.

[19] Mao F., Hong J., Min Q., Gong W., Zang L., Yin J. (2021). Estimating hourly full-coverage PM2.5 over China based on TOA reflectance data from the Fengyun-4A satellite. Environ. Pollut..

[20] Wei J., Li Z., Lyapustin A., Sun L., Peng Y., Xue W., Su T., Cribb M. (2021). Reconstructing 1-km-resolution high-quality PM2.5 data records from 2000 to 2018 in China: Spatiotemporal variations and policy implications. Remote Sens. Environ..

[21] Wei N., Jia Z., Men Z., Ren C., Zhang Y., Peng J., Wu L., Wang T., Zhang Q., Mao H. (2022). Machine Learning Predicts Emissions of Brake Wear PM2.5: Model Construction and Interpretation. Environ. Sci. Technol. Lett..

[22] Wang Z., Li R., Chen Z., Yao Q., Gao B., Xu M., Yang L., Li M., Zhou C. (2022). The estimation of hourly PM2.5 concentrations across China based on a Spatial and Temporal Weighted Continuous Deep Neural Network (STWC-DNN). ISPRS J. Photogramm. Remote Sens..

[23] Zou G., Zhang B., Yong R., Qin D., Zhao Q. (2021). FDN-learning: Urban PM2.5-concentration Spatial Correlation Prediction Model Based on Fusion Deep Neural Network. Big Data Res..

[24] Rybarczyk Y., Zalakeviciute R. Machine learning approach to forecasting urban pollution. Proceedings of the 2016 IEEE Ecuador Technical Chapters Meeting (ETCM).

[25] Gore R.W., Deshpande D.S. An approach for classification of health risks based on air quality levels. Proceedings of the 2017 1st International Conference on Intelligent Systems and Information Management (ICISIM).

[26] Di Q., Amini H., Shi L., Kloog I., Silvern R., Kelly J., Sabath M.B., Choirat C., Koutrakis P., Lyapustin A. (2019). An ensemble-based model of PM2.5 concentration across the contiguous United States with high spatiotemporal resolution. Environ. Int..

[27] Kumar S., Mishra S., Singh S.K. (2020). A machine learning-based model to estimate PM2.5 concentration levels in Delhi’s atmosphere. Heliyon.

[28] Feng C., Wang W., Tian Y., Que X., Gong X. Estimate air quality based on mobile crowd sensing and big data. Proceedings of the 2017 IEEE 18th International Symposium on a World of Wireless, Mobile and Multimedia Networks (WoWMoM).

[29] Kleine Deters J., Zalakeviciute R., Gonzalez M., Rybarczyk Y. (2017). Modeling PM2.5 urban pollution using machine learning and selected meteorological parameters. J. Electr. Comput. Eng..

[30] Wang P., Zhang H., Qin Z., Zhang G. (2017). A novel hybrid-Garch model based on ARIMA and SVM for PM2.5 concentrations forecasting. Atmos. Pollut. Res..

[31] Yarragunta S., Nabi M.A. Prediction of air pollutants using supervised machine learning. Proceedings of the 2021 5th International Conference on Intelligent Computing and Control Systems (ICICCS).

[32] Goudarzi G., Hopke P.K., Yazdani M. (2021). Forecasting PM2.5 concentration using artificial neural network and its health effects in Ahvaz, Iran. Chemosphere.

[33] Feng X., Li Q., Zhu Y., Hou J., Jin L., Wang J. (2015). Artificial neural networks forecasting of PM2.5 pollution using air mass trajectory based geographic model and wavelet transformation. Atmos. Environ..

[34] Choi S.-M., Choi H. (2022). Artificial Neural Network Modeling on PM10, PM2.5, and NO2 Concentrations between Two Megacities without a Lockdown in Korea, for the COVID-19 Pandemic Period of 2020. Int. J. Environ. Res. Public Health.

[35] Chae S., Shin J., Kwon S., Lee S., Kang S., Lee D. (2021). PM10 and PM2.5 real-time prediction models using an interpolated convolutional neural network. Sci. Rep..

[36] Chakma A., Vizena B., Cao T., Lin J., Zhang J. Image-based air quality analysis using deep convolutional neural network. Proceedings of the 2017 IEEE International Conference on Image Processing (ICIP).

[37] Li J., Jin M., Li H. (2019). Exploring spatial influence of remotely sensed PM2.5 concentration using a developed deep convolutional neural network model. Int. J. Environ. Res. Public Health.

[38] Tong W., Li L., Zhou X., Hamilton A., Zhang K. (2019). Deep learning PM2.5 concentrations with bidirectional LSTM RNN. Air Qual. Atmos. Health.

[39] Tsai Y.-T., Zeng Y.-R., Chang Y.-S. Air pollution forecasting using RNN with LSTM. Proceedings of the 2018 IEEE 16th Intl Conf on Dependable, Autonomic and Secure Computing, 16th Intl Conf on Pervasive Intelligence and Computing, 4th Intl Conf on Big Data Intelligence and Computing and Cyber Science and Technology Congress (DASC/PiCom/DataCom/CyberSciTech).

[40] Liu B., Yan S., Li J., Li Y., Lang J., Qu G. (2021). A spatiotemporal recurrent neural network for prediction of atmospheric PM2.5: A case study of Beijing. IEEE Trans. Comput. Soc. Syst..

[41] Gao X., Li W. (2021). A graph-based LSTM model for PM2.5 forecasting. Atmos. Pollut. Res..

[42] Qadeer K., Rehman W.U., Sheri A.M., Park I., Kim H.K., Jeon M. (2020). A long short-term memory (LSTM) network for hourly estimation of PM2.5 concentration in two cities of South Korea. Appl. Sci..

[43] Zhang M., Wu D., Xue R. (2021). Hourly prediction of PM2.5 concentration in Beijing based on Bi-LSTM neural network. Multimed. Tools Appl..

[44] Babu S., Thomas B. (2021). A recurrent neural network forecasting technique for daily PM2.5 concentration level in Southern Kerala. IOP Conference Series: Materials Science and Engineering.

[45] Wei P., Xie S., Huang L., Zhu G., Tang Y., Zhang Y. (2021). Prediction of PM2.5 concentration in Guangxi region, China based on MLR-ARIMA. J. Phys. Conf. Ser..

[46] Teng M., Li S., Xing J., Song G., Yang J., Dong J., Zeng X., Qin Y. (2022). 24-Hour prediction of PM2.5 concentrations by combining empirical mode decomposition and bidirectional long short-term memory neural network. Sci. Total Environ..

[47] Jin X.-B., Yang N.-X., Wang X.-Y., Bai Y.-T., Su T.-L., Kong J.-L. (2020). Deep hybrid model based on EMD with classification by frequency characteristics for long-term air quality prediction. Mathematics.

[48] Zhao J., Deng F., Cai Y., Chen J. (2019). Long short-term memory-Fully connected (LSTM-FC) neural network for PM2.5 concentration prediction. Chemosphere.

[49] Dai H., Huang G., Zeng H., Yang F. (2021). PM2.5 Concentration Prediction Based on Spatiotemporal Feature Selection Using XGBoost-MSCNN-GA-LSTM. Sustainability.

[50] Li T., Hua M., Wu X. (2020). A hybrid CNN-LSTM model for forecasting particulate matter (PM2.5). IEEE Access.

[51] Huang C.-J., Kuo P.-H. (2018). A deep CNN-LSTM model for particulate matter (PM2.5) forecasting in smart cities. Sensors.

[52] Qin D., Yu J., Zou G., Yong R., Zhao Q., Zhang B. (2019). A novel combined prediction scheme based on CNN and LSTM for urban PM 2.5 concentration. IEEE Access.

[53] Zhu M., Xie J. (2023). Investigation of nearby monitoring station for hourly PM2.5 forecasting using parallel multi-input 1D-CNN-biLSTM. Expert Syst. Appl..

[54] Zhang J., Peng Y., Ren B., Li T. (2021). PM2.5 Concentration Prediction Based on CNN-BiLSTM and Attention Mechanism. Algorithms.

[55] Chen Y., Ye C., Yang P., Miao Z., Chen Y., Li H., Liu R., Liu B. Research on An Attention-based Hybrid CNN and BiLSTM Model for Air Pollutant Concentration Prediction. Proceedings of the 2021 6th International Conference on Computational Intelligence and Applications (ICCIA).

[56] Wong P.-Y., Lee H.-Y., Chen Y.-C., Zeng Y.-T., Chern Y.-R., Chen N.-T., Lung S.-C.C., Su H.-J., Wu C.-D. (2021). Using a land use regression model with machine learning to estimate ground level PM2.5. Environ. Pollut..

[57] Wang X., Sun W., Zheng K., Ren X., Han P. (2020). Estimating hourly PM2.5 concentrations using MODIS 3 km AOD and an improved spatiotemporal model over Beijing-Tianjin-Hebei, China. Atmos. Environ..

[58] Shen J., Valagolam D., McCalla S. (2020). Prophet forecasting model: A machine learning approach to predict the concentration of air pollutants (PM2.5, PM10, O3, NO2, SO2, CO) in Seoul, South Korea. PeerJ.

[59] Li X., Zhang X. (2019). Predicting ground-level PM2.5 concentrations in the Beijing-Tianjin-Hebei region: A hybrid remote sensing and machine learning approach. Environ. Pollut..

[60] Ejohwomu O.A., Shamsideen Oshodi O., Oladokun M., Bukoye O.T., Emekwuru N., Sotunbo A., Adenuga O. (2022). Modelling and forecasting temporal PM2.5 concentration using ensemble machine learning methods. Buildings.

